# Cirsiliol Suppressed Epithelial to Mesenchymal Transition in B16F10 Malignant Melanoma Cells through Alteration of the PI3K/Akt/NF-κB Signaling Pathway

**DOI:** 10.3390/ijms20030608

**Published:** 2019-01-31

**Authors:** Priyanka Prasad, Andrea Vasas, Judit Hohmann, Anupam Bishayee, Dona Sinha

**Affiliations:** 1 Receptor Biology and Tumor Metastasis, Chittaranjan National Cancer Institute, Kolkata 700 026, India; pppinki025@gmail.com; 2 Department of Pharmacognosy, University of Szeged, Eötvös u. 6, H-6720 Szeged, Hungary; vasasa@pharm.u-szeged.hu (A.V.); hohmann@pharm.u-szeged.hu (J.H.); 3 Interdisciplinary Centre for Natural Products, University of Szeged, Eötvös u. 6, H-6720 Szeged, Hungary; 4 Lake Erie College of Osteopathic Medicine, Bradenton, FL 34211, USA

**Keywords:** melanoma, *Centaurea jacea*, cirsiliol, EMT, cadherin, MMP-9, PI3K, Akt, NF-κB

## Abstract

Malignant melanoma is a highly aggressive form of skin cancer which has a propensity for metastasis. Epithelial mesenchymal transition (EMT) plays a primordial role in the progression of metastatic disease. Metastatic melanoma is resistant to conventional therapies. Hence, researchers have been exploring alternative approaches, including the utility of bioactive phytochemicals to manage metastatic disease. In the present study, we investigated the potential of cirsiliol, a flavonoid isolated from *Centaurea jacea* L., in modulating the aggressive behavior of B16F10 metastatic melanoma cells, including EMT, and associated molecular mechanisms of action. Cirsiliol was found to be effective in restraining the colony formation and migration of fibronectin-induced B16F10 metastatic melanoma cells. Cirsiliol inhibited the activity and expression of matrix metalloproteinase-9 (MMP-9). Cirsiliol also suppressed the phosphatidylinositol-3-kinase (PI3K)/protein kinase B (also known as Akt)/nuclear factor-κB (NF-κB) signaling pathway which, in turn, caused upregulation of E-cadherin and downregulation of N-cadherin, Snail and Twist. Based on these results, cirsiliol may be considered a promising compound against EMT in the therapeutic management of malignant melanoma.

## 1. Introduction

Malignant melanoma is the most aggressive form of skin cancer. About 91,270 new incidences of melanoma (55,150 in men and 36,120 in women) and 9320 deaths (5990 men and 3330 women) due to this disease are estimated to occur in the United States in 2018 [1]. The rates of melanoma have been rising for the last 30 years. It is represented by an overall mortality rate of around 20% and is responsible for 80% of global skin cancer-related deaths [2]. Early-stage melanoma can be effectively treated with surgery, but metastatic melanoma is highly resistant to conventional therapies [3]. The high rate of mortality is related to its chemotherapy resistance, aggressive behavior, and propensity to metastasize rapidly [4].

Melanoma is not of epithelial origin, rather it develops from neural crest-derived pigmented melanocytes. However, the hallmarks of epithelial-to-mesenchymal transition (EMT) play critical roles in its progression [5]. EMT is a dynamic and reversible process where polarized epithelial cells transform into motile mesenchymal cells and is propagated to be a critical event for tumor cell invasiveness [6,7]. Differentiated melanocytes express epithelial (E)cadherin, which is required for their association with keratinocytes in the basal epidermal layer. Loss of Ecadherin represents a hallmark of EMT in epithelial tumors which is also evident during nodal metastases in malignant melanoma [8]. EMT-inducing transcription factors, including Snail, Twist, and zinc finger Ebox (ZEB), play important roles in malignant melanoma, but their regulation and function are different from those in epithelial cancers [8,9]. The phosphatidylinositol-3-kinase (PI3K) pathway is known to be crucially involved in melanoma [2], and this pathway plays a significant role in EMT [10]. Ras transcription is associated with positive regulation of the PI3K/protein kinase B (Akt) signaling pathway, which leads to activation of Akt and its downstream targets. Activation of PI3K/Akt pathway causes cell proliferation, dissemination, and survival of cancer cells [11]. PI3K/Akt signaling aggravated transforming growth factor-β (TGF-β)-induced EMT in A549 lung cancer cells [12].

At present, there is no effective treatment for metastatic melanoma, and five-year survival does not exceed 15% in patients with metastatic disease. Hence, researchers have focused on effective compounds from natural sources to manage metastatic disease. In this regard, the role of many natural products and phytochemicals in regulation of EMT in various cancer models through attenuation of primary signaling pathways has been established [13]. The flavonoids are an important group of plant secondary metabolites that include varied classes of polyphenolic compounds found in fruits, vegetables, roots, stems, flowers, beans and beverages, including tea and wine. Flavonoids are well known for their multi-targeted anticancer properties, which include induction of apoptosis as well as inhibition of cell proliferation, angiogenesis, and metastasis [14]. Flavonoids, such as quercetin and luteolin, have shown efficacy against EMT in A431 melanoma cells [15]. Cirsiliol (5,3’,4’-trihydroxy-6,7-dimethoxyflavone) is a flavonoid belonging to the class flavone. It was first isolated from the desert plant *Achillea fragrantissima* [16]. Later, it was also found in other sources, such as chloroform extract of the aerial parts of *Centaurea jacea* L. [17], epicuticular wax of the leaves of *Arabidea brachipoda* [18] and ethanolic extract of the aerial part of *Artemisia scoparia* [19]. Emerging studies with cirsiliol revealed several therapeutic properties, such as anti-infective (against human immunodeficiency virus, hepatitis C virus and toxoplasmosis), anti-obesity and anti-fungal activities [18,19,20]. Cirsiliol was found to exhibit cell growth-inhibitory activities against various cancer cells, such as HeLa, MCF-7 and A431 cells [17]. Cirsiliol along with rhamnetin restrained EMT and radio-resistance in non-small cell lung cancer cell lines, NCI-H1299 and NCI-H460, by inhibiting the overexpression of Notch 1 [21]. Moreover, cirsiliol exhibited antiproliferative activity by inhibiting arachidonate-5-lipooxygenase in human leukemic cell lines, such as K562, Molt-4B and HL-60 [22]. Nevertheless, therapeutic potential of cirsiliol against metastatic melanoma has not been studied yet as per our knowledge. Accordingly, the present study was aimed to investigate the potential of cirsiliol in modulating the aggressive behavior of metastatic melanoma cells, such as EMT, and associated molecular mechanisms of action.

## 2. Results

### 2.1. Effects of Cirsiliol on Mortality, Colony Formation and Cell Cycle of Metastatic Melanoma Cells

**Figure 1:**
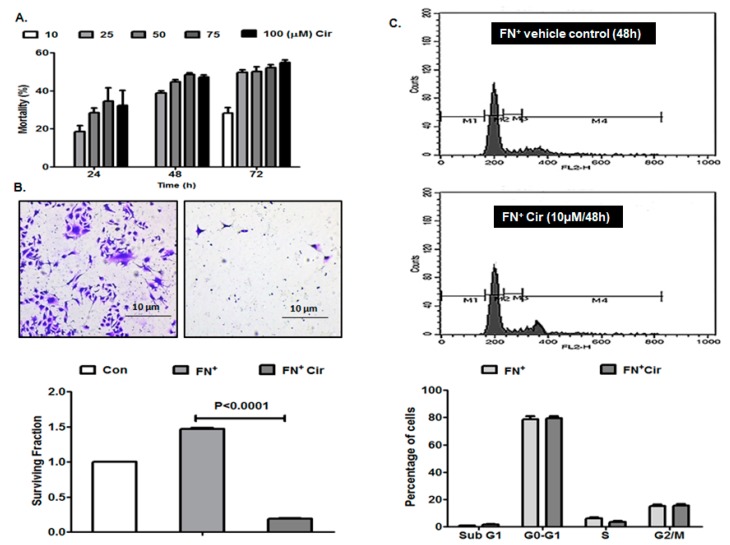
Effects of cirsiliol on cell mortality, colony formation and cell cycle of B16F10 cells. (**A**) Concentration- and time-dependent cytotoxic effect of cirsiliol. (**B**) Colony formation assay micrographs (400× magnification) and graphical representation of significant inhibition of surviving fraction in fibronectin (FN^+^) and cirsiliol (Cir) [10 µM/48 h]-treated cells compared to cells exposed to FN only. (**C**) No significant alteration of percentage of cells in different phases of cell cycle was observed between FN^+^/Cir (10 µM/48 h) cells and FN-induced cells treated with vehicle as depicted by representative figure and graph. All quantitative results are expressed as mean ± standard deviation (SD) based on three replicates. M1: Sub G1; M2: G0-G1; M3: S; and M4: G2/M.

MTT assay conducted for evaluating the effect of cirsiliol on the mortality of B16F10 metastatic melanoma cells revealed that treatment with this phytochemical at a concentration of 10 µM for 24 h or 48 h did not induce any mortality. The vehicle dimethyl sulfoxide (DMSO) (0.01%) did not have any effect on the viability of B16F10 cells. Cirsiliol at 10 µM induced 28% mortality of B16F10 cells only after 72 h (Figure 1A). A 50% inhibitory concentration(IC_50_) of cirsiliol could not be achieved at 24 h or 48 h. Even cirsiliol (50 µM) after 48 h caused 44% mortality in B16F10 cells after which a plateau was achieved. In case of 72 h treatment, IC_50_ of cirsiliol was found to be 25 µM. Cirsiliol at 10 µM for 48 h was also nontoxic for HaCaT normal skin keratinocytes (data not shown). Hence, the non-cytotoxic concentration of cirsiliol (10 µM) for 48 h treatment period was used for subsequent studies.

Colony formation assay exhibited significant inhibition of survival of fibronectin (FN)-induced and cirsiliol (10 µM/48 h)-treated B16F10 cells compared to B16F10 cells exposed to FN only (Figure 1B).

No significant alteration of percentage of B16F10 cells in different phases of cell cycle was observed between FN-induced and cirsiliol (10 µM/48 h)-treated cells and FN-induced B16F10 cells treated with vehicle (Figure 1C).

### 2.2. Cirsiliol Inhibited Migratory Potential of FN-Induced Melanoma Cells 

**Figure 2:**
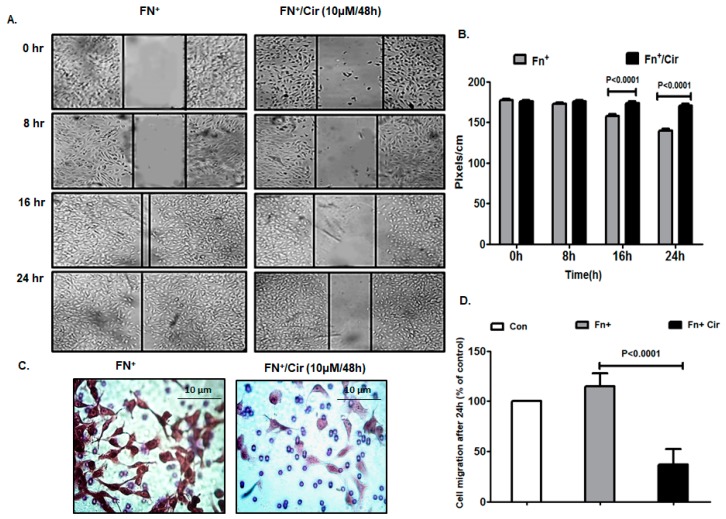
Effect of cirsiliol on migratory potential of B16F10 cells. (**A**) Wound healing assay showed reduction in the migratory property of FN^+^/Cir (10 µM/48 h)-treated B16F10 cells with respect to fibronectin (FN^+^) [20 µg/mL]-induced cells even after 24 h of monitoring. (**B**) The distance of wound closure (measured by image J software in pixels/cm) was much more in case of FN^+^/Cir (10 µM/48 h) treated B16F10 cells than FN^+^-induced cells. (**C**) Trans-well migration assay micrographs (400× magnification) showed compromised migratory property in FN^+^/Cir (10 µM/48 h)-treated B16F10 cells compared to FN^+^ B16F10 cells. (**D**) The percentage of cells which migrated across the Boyden’s chamber were significantly less in case of FN^+^/Cir (10 µM/48 h)-treated cells than the FN^+^-induced cells. Cir: cirsiliol; FN^+^: cells were seeded in pre-coated (cellular FN 20 μg/mL for 2 h at 37 °C) petri dishes and further incubated for 48 h in presence of 5% fetal bovine serum (FBS) for induction of EMT; FN^+^/Cir (10 µM/48 h): cells were seeded in pre-coated (cellular FN 20 μg/mL for 2 h at 37 °C) petri dishes and subjected to a non-cytotoxic dose of cirsiliol (10 µM) treatment for 48 h. All quantitative results are expressed as mean ± SD based on three replicates.

Cell migration is the key to embryonic development, wound healing and cancer metastasis by inducing EMT which is highly conserved transitional program characterized by alterations at morphological, structural and molecular levels [23]. Thus, we assessed the effect of cirsiliol on the migratory potential of FN-induced B16F10 cells by wound healing assay. The results exhibited slow healing of the wound/scratch in the monolayer of B16F10 cells treated with cirsiliol (10 µM/48 h) in comparison to those treated only with FN (Figure 2A). By the end of 16 or 24 h, the wound closure was significantly inhibited by cirsiliol (10 µM/48 h) in FN-induced cells (Figure 2B). This was further validated by trans-well migration assay where cirsiliol (10 µM/48 h) inhibited the migration of FN-induced cells by 80% (Figure 2C,D).

### 2.3. Cirsiliol Reduced Matrix Metalloprotienase-9 (MMP-9) Activity and Expression in FN-Induced Cells

**Figure 3:**
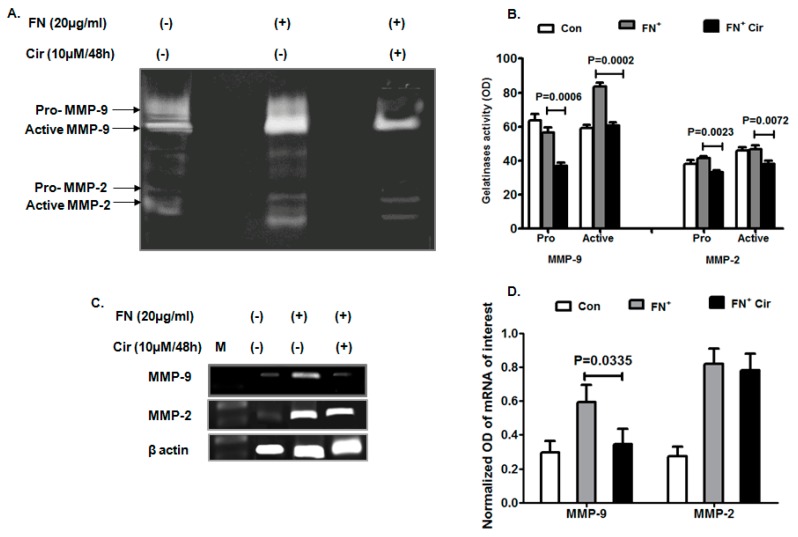
Effect of cirsiliol on gelatinases of B16F10 cells. (**A**) Representative zymogram elicited that FN-induced increase in activity of active MMP-2 and MMP-9 was significantly decreased when the cells were treated with FN^+^/Cir (10 µM/48 h). (**B**) Mean band intensities showed that FN^+^/Cir (10 µM/48 h) significantly inhibited activity of both pro and active forms of MMP-2 and MMP-9 than FN^+^- treated B16F10 cells. (**C**) Inhibition of MMP-9 expression by FN^+^/Cir (10 µM/48 h) with respect to FN^+^-induced cells. On the other hand, transcript profile of MMP-2 did not show any significant change of expression by FN^+^/Cir (10 µM/48 h) with respect to FN^+^-induced cells. (**D**) FN^+^/Cir (10 µM/48 h) caused significant inhibition of mean MMP-9 mRNA expression and no change of mean MMP-2 mRNA expression in comparison to only FN-induced cells. Cir: cirsiliol; FN^+^: cells were seeded in pre-coated (cellular FN 20 μg/mL for 2 h at 37 °C) petri dishes and further incubated for 48 h in presence of 5% FBS for induction of EMT; FN^+^/Cir (10 µM/48 h): cells were seeded in pre-coated (cellular FN 20 μg/mL for 2 h at 37 °C) petri dishes and treated with subjected to a non-cytotoxic dose of cirsiliol (10 µM) treatment for 48 h; M: DNA step ladder 100 bp. All quantitative results are expressed as mean ± SD based on three replicates.

Remodeling of cancer cells is important for migration, invasion and metastasis in which matrix metalloproteinases play a vital role. Gelatinases are important for tissue degradation and cellular infiltration. MMP-2 plays a significant role in angiogenesis and tumor formation, while MMP-9 is vital for metastasis and tumor development. Both pro and active form of MMP-2 and MMP-9 were significantly reduced with cirsiliol (10 µM/48 h) in FN-induced cells as evident from the zymography results (Figure 3A,B). The transcript profile also exhibited suppression of MMP-9 expression by cirsiliol (10 µM/48 h) in FN^+^-induced cells (Figure 3C,D). However, MMP-2 did not show any change of expression in the presence or absence of cirsiliol (Figure 3C,D).

### 2.4. Cirsiliol Altered Expression of Cadherins in FN-Induced B16F10 Cells

**Figure 4:**
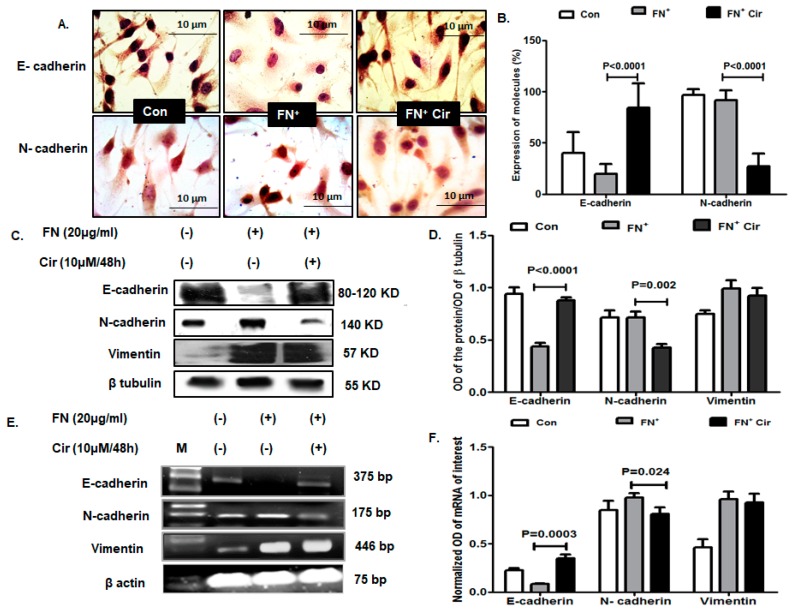
Effect of cirsiliol on epithelial and mesenchymal markers in B16F10 cells. (**A**) Representative micrographs of immunocytochemistry (400× magnification) and (**B**) graph with percentage of cells expressing E-cadherin and N-cadherin revealed that FN^+^/Cir (10 µM/48 h) increased cytoplasmic expression of E-cadherin and decreased that of N-cadherin with respect to FN^+^-induced cells. (**C**) Images of western blot and (**D**) quantitative expression of mean band intensity exhibited that FN-induced downregulation of E-cadherin and upregulation of N-cadherin was significantly reversed with FN^+^/Cir (10 µM/48 h). Vimentin did not show any significant change of expression by FN^+^/Cir (10 µM/48 h) with respect to FN^+^-induced cells. (**E**) The transcript profile images and the (**F**) mean band intensities showed that FN^+^-induced downregulation of E-cadherin and upregulation of N-cadherin was significantly counteracted with FN^+^/Cir (10 µM/48 h). Vimentin did not show any significant change of mRNA expression by FN^+^/Cir (10 µM/48 h) in comparison to FN^+^-induced cells. Cir: cirsiliol; FN^+^: cells were seeded in pre-coated (cellular FN 20 μg/mL for 2 h at 37 °C) petri dishes and further incubated for 48 h in presence of 5% FBS for induction of EMT; FN^+^/Cir (10 µM/48 h): cells were seeded in pre-coated (cellular FN 20 μg/mL for 2 h at 37 °C) petri dishes and treated with subjected to a non-cytotoxic dose of cirsiliol (10 µM) treatment for 48 h; M: DNA step ladder 100 bp. All quantitative results are expressed as mean ± SD based on three replicates. Scanned blots of Figure 4 are showed in Figure S1.

EMT alters the cell to cell adhesion receptors by downregulation of the E-cadherin and upregulation of neural (N)-cadherin. With initiation of EMT, the cell to cell adhesion junctions are lost and the proteins associated are either degraded or relocated [24]. Based on immunocytochemical analysis, FN-induced melanoma cells elicited downregulation of E-cadherin and upregulation of N-cadherin compared to control cells (Figure 4A,B). Following the treatment of the FN-induced cells with cirsiliol (10 µM/48 h), the cellular expression of E-cadherin was upregulated by 4.3-fold and that of N-cadherin was downregulated by 3.4-fold (Figure 4A,B). Western blot analysis reflected similar trend of E-cadherin and N-cadherin expression in FN^+^/cirsiliol (10 µM/48 h) with respect to FN^+^ cells as depicted in the representative image (Figure 4C) and mean band intensity (Figure 4D). These findings were confirmed at the transcriptional level as shown with representative images (Figure 4E) and mean band intensities of mRNA (Figure 4F). However, vimentin expression did not show any significant change in either protein (Figure 4C,D) or mRNA level (Figure 4E,F) between FN^+^/cirsiloil (10 µM/48 h) cells and FN^+^ melanoma cells. 

### 2.5. Cirsiliol Reduced FN-Induced EMT Transcription Factors in B16F10 Cells

**Figure 5:**
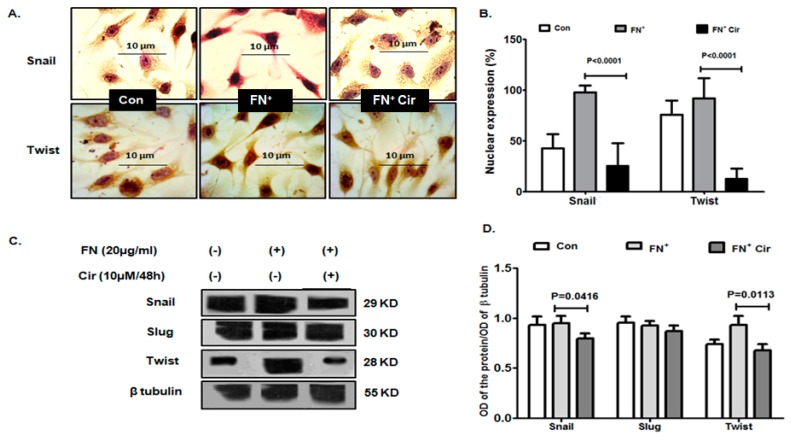
Effect of cirsiliol on the EMT transcription factors of B16F10 cells. (**A**) Representative micrographs of immunocytochemistry (400× magnification) and (**B**) quantitative analysis of cells indicated reduced nuclear expression of Snail and Twist in FN^+^/Cir (10 µM/48 h)-treated cells compared to FN^+^-induced cells. (**C**) Representative images of western blot and (**D**) the normalized mean band intensity graphs indicated reduced expression of Snail and Twist in FN^+^/Cir (10 µM/48 h) treated cells in comparison with FN^+^-induced cells. Cir: cirsiliol; FN^+^: cells were seeded in pre-coated (cellular FN 20 μg/mL for 2 h at 37 °C) petri dishes and further incubated for 48 h in presence of 5% FBS for induction of EMT; FN^+^/Cir (10 µM/48 h): cells were seeded in pre-coated (cellular FN 20 μg/mL for 2 h at 37 °C) petri dishes and treated with subjected to a non-cytotoxic dose of cirsiliol (10 µM) treatment for 48 h. All quantitative results are expressed as mean ± SD based on three replicates. Scanned blots of Figure 5 are showed in Figure S2.

The mesenchymal characteristics in a cell are induced by master regulatory transcription factors, such as Snail, Slug and Twist. They control the expression of E-cadherin as well as other mesenchymal phenotypes, including activation MMP expression [25]. Immunocytochemistry revealed that cirsiliol (10 µM/48 h) reduced nuclear localization of both Snail and Twist in the FN-treated cells that had increased after EMT induction with FN (Figure 5A). The percentage of cells expressing nuclear Snail were decreased by 3.9-fold and that of Twist was decreased by 7.3-fold in case of cells treated with FN^+^/cirsiliol (10 µM/48 h) compared to FN^+^ melanoma cells (Figure 5B). The western blot results exhibited the similar effect of cirsiliol (10 µM/48 h) to reduce the expression of Snail and Twist in FN-induced cells (Figure 5C). However, cirsiliol did not exhibit any effect on Slug (Figure 5C). The mean band intensities reflected similar modulation of Snail, Twist and Slug by FN+/cirsiliol (10 µM/48 h) with respect to FN^+^ B16F10 cells (Figure 5D).

### 2.6. Cirsiliol Suppressed PI3K/Akt Signaling in FN-Induced B16F10 Cells

**Figure 6:**
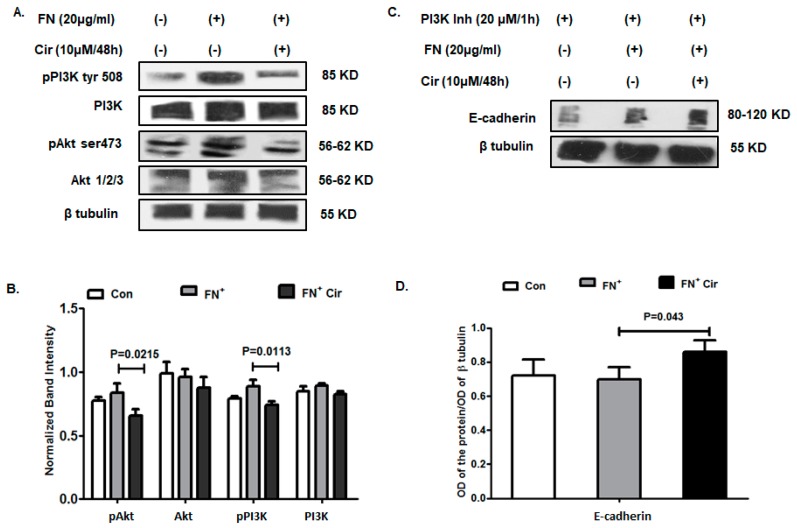
Effect of cirsiliol on regulation of EMT in B16F10 cells. (**A**) Representative images of western blot and (**B**) graphical representation of the normalized band intensities implied reduced phosphorylation status of both PI3Ktyr508 and pAktser473 treatment with FN^+^/Cir (10 µM/48 h) treated cells than FN^+^-induced cells. (**C**) Western blot images and (**D**) graphical representation of mean band intensities of the upregulated expression of E-cadherin in the FN^+^-induced B16F10 cells and FN^+^/Cir (10 µM/48 h) treated B16F10 cells in comparison to control cells in presence of PI3K inhibitor. Cir: cirsiliol; FN^+^: cells were seeded in pre-coated (cellular FN 20 μg/mL for 2 h at 37 °C) petri dishes and further incubated for 48 h in the presence of 5% FBS for induction of EMT; FN^+^/Cir (10 µM/48 h): cells were seeded in pre-coated (cellular FN 20 μg/mL for 2 h at 37 °C) petri dishes and treated with subjected to a non-cytotoxic dose of cirsiliol (10 µM) treatment for 48 h. All quantitative results are expressed as mean ± SD based on three replicates. Scanned blots of Figure 6 are showed in Figure S3.

The PI3K/Akt signaling plays an important role in regulating the mechanism of EMT by affecting a variety of cellular and physiological processes. On one hand, Akt regulates the functioning of Twist by phosphorylation that again acts in a positive feedback loop with Twist to enhance the pro-EMT function while on the other hand, it prevents degradation of Snail as well as enhancing its intracellular expression [26,27]. It has also been found to enhance the expression of MMP-9 that remodels tissue and aids in invasion. The current investigation of the expression of these regulatory molecules revealed a significant reduction in phosphorylation status of PI3K at tyrosine residue 508 and Akt at serine 473 by FN^+^/cirsiliol (10 µM/48 h) in comparison to FN-induced B16F10 cells as evident from the western blot (Figure 6A) and the mean band intensities (Figure 6B). To further validate the mechanistic role of cirsiliol (10 µM/48 h) through PI3K, cells were treated with PI3K inhibitor followed by induction with FN. Analysis of the E-cadherin expression by western blot revealed elevation in its expression in the case of FN^+^ cells with respect to the control. The expression of E-cadherin was elevated with FN^+^/cirsiliol (10 µM/48 h) in comparison to FN^+^ cells (Figure 6C). The mean band intensities of E-cadherin also reflected a similar pattern of upregulation in the presence of PI3K inhibitor with FN^+^ cells and FN^+^/cirsiliol (10 µM/48 h) treated cells (Figure 6D). This validated the finding that cirsiliol probably acted by inhibiting the PI3K/Akt pathway against FN-induced EMT in B16F10 cells.

### 2.7. Cirsiloil Suppressed Nuclear Factor-κB p65 (NF-κB p65) Signaling in FN-Induced B16F10 Cells

**Figure 7:**
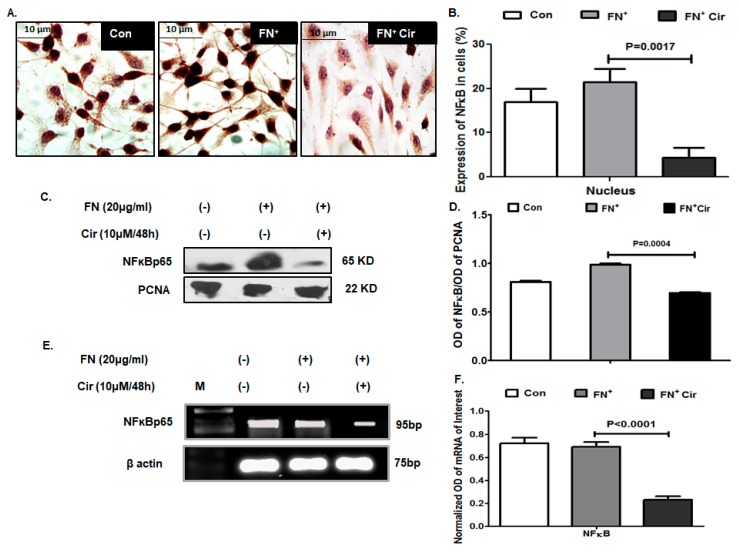
Effect of cirsiliol on NF-κB signaling in B16F10 cells. (**A**) Micrographs of immunocytochemistry (400× magnification) exhibited reduced nuclear expression of NF-κB p65 in FN^+^/Cir (10 µM/48 h) treated cells than the FN^+^-induced cells. (**B**) Quantitative analysis of cells with positive nuclear expression of NF-κB p65. (**C**) Western blot images and (**D**) graphical representation of the normalized band intensities of nuclear NF-κB p65 in FN^+^/Cir (10 µM/48 h) treated cells in comparison to FN^+^ B16F10 cells. (**E**) mRNA images and (**F**) mean band intensities of mRNA of NF-κB p65 in FN^+^/Cir (10 µM/48 h)-treated cells in comparison to FN^+^ B16F10 cells. Cir: cirsiliol; FN^+^: cells were seeded in pre-coated (cellular FN 20 μg/mL for 2 h at 37 °C) petri dishes and further incubated for 48 h in presence of 5% FBS for induction of EMT; FN^+^/Cir (10 µM/48 h): cells were seeded in pre-coated (cellular FN 20 μg/mL for 2 h at 37 °C) petri dishes and subjected to a non-cytotoxic dose of cirsiliol (10 µM) treatment for 48 h, M: DNA step ladder 100 bp. All quantitative results are expressed as mean ± SD based on three replicates. Scanned blots of Figure 7 are showed in Figure S4.

The PI3K/Akt pathway also plays a major role in activation of NF-κB p65 subunit that in turn is associated with the induction of cellular expression of Snail [28]. Immunocytochemical analysis revealed that cirsiliol (10 µM/48 h) treatment reduced the nuclear localization of NF-κB p65 in FN-induced B16F10 cells in comparison with FN^+^ cells (Figure 7A). The percentage of cells expressing nuclear NF-κB p65 decreased with FN^+^/cirsiliol (10 µM/48 h) compared to FN^+^ cells (Figure 7B). The expression of nuclear NF-κB p65 was downregulated with FN^+^/cirsiliol (10 µM/48 h) in comparison with FN^+^ cells as evident from western blot results (Figure 7C,D). Similar results were observed with the transcriptional profile of NF-κB p65 (Figure 7E,F).

## 3. Discussion

EMT is the switching of epithelial cells to mesenchymal-like cells by loss of apical-basal polarity and cell-cell adhesion, switch of cell surface markers and enhanced migratory and invasive capacities [23]. The molecular mechanism of EMT is associated with the downregulation of E-cadherin and upregulation of mesenchymal markers, such as N-cadherin, fibronectin and vimentin [29]. E-cadherin transcription is inhibited by transcription factors, such as ZEB, Snail and Twist [30].

Phytochemicals have been found to possess numerous health-promoting and disease-preventive activities. Since there is no effective treatment against metastatic melanoma, various phytochemicals have been widely explored for their antitumor and antimetastatic potential. *C. jacea* L. (also known as brown knapweed or brownray knapweed) is a perennial herb with widespread distribution in Europe. The flavone cirsiliol was isolated from the chloroform extract of *C. jacea* L. and it showed antiproliferative activity against HeLa, MCF-7 and A431 tumor cell lines [17]. Cirsiliol exhibited growth-inhibitory activity against MCF-7 breast cancer cells by inducing cytochrome P450 family 1 enzyme [31]. Thus, antiproliferative efficacy of cirsiliol against tumor cell lines had been well explored, but its antimetastatic potential has not been investigated before as per our knowledge.

Melanoma is characterized by EMT-mediated tumor progression [5]. Induction of EMT by TGF-β is a well-established phenomenon. A non-cytotoxic concentration of cirsiliol (10 µM/48 h) was effective in reducing the migratory properties of B16F10 cells, which prompted us to investigate its role against EMT in melanoma cells. FN is one of the prime components of extracellular matrix which is well-known for regulating normal cell adhesion and migration. Over-expression of FN, reported in several solid carcinomas, has been propagated to be beneficial for establishment of an ideal microenvironment for tumor growth [32]. In the present study, we tried to explore the induction of EMT with extracellular matrix (ECM) component FN. Since “cadherin switch” characterized by downregulation of E-cadherin and upregulation of N-cadherin is regarded as the hallmark of EMT, we investigated the status of E-cadherin and N-cadherin following FN treatment. FN-stimulated melanoma cells exhibited robust downregulation of E-cadherin and upregulation of N-cadherin. Interestingly, the non-cytotoxic concentration of cirsiliol was found to reverse this cadherin switch. Cirsiliol upregulated E-cadherin expression and downregulated N-cadherin expression in FN-induced B16F10 melanoma cells. This was in congruence with previous studies where cirsiliol along with rhamnetin downregulated Notch-1-mediated radio-resistance and EMT phenotypes in non-small cell lung carcinoma cell lines [21].

The occurrence of EMT is associated with several complex signal transduction pathways, including the PI3K/Akt signaling pathway [33]. The PI3K/Akt pathway, crucially involved in metastatic melanoma [34], is responsible for cell proliferation, anabolism, dissemination, and tumor survival [2]. The flavonoid fisetin impeded EMT in A375 melanoma cells by hindering the PI3K/Akt/mammalian target of rapamycin (mTOR) pathway [35]. ECM component FN was reported to be associated with PI3K/Akt-mediated metastasis and cancer cell resistance to apoptosis [36]. In the present study, FN substantially elevated the expression of pPI3KTyr508 and pAKTSer473 in melanoma cells which were suppressed by cirsiliol treatment. Moreover, it was observed that with the use of PI3K inhibitor, E-cadherin expression increased which was further elevated in the presence of cirsiliol. This clearly indicated that suppression of E-cadherin which is the hallmark of EMT progression, might have been achieved through downregulation of PI3K/Akt pathway.

The EMT transcription factors, such as Snail and Slug, can shuttle between the cytoplasm and the nucleus in order to bind E-box promoter of E-cadherin, inhibit its expression and activate EMT [37]. The PI3K/Akt signaling pathway on one hand, prevents glycogen synthase kinase 3β (GSK3β)-mediated degradation of Snail and on the other hand, directly activates expression of Snail [27]. In accordance with the above studies, FN-induced B16F10 cells exhibited that phosphorylation of PI3K at Tyr508 and Akt at Ser473 caused upregulation of Snail and downregulation of E-cadherin, and that cirsiliol was effective in counteracting these effects. The nuclear localization of Snail influences the transcription of β-catenin and also increases the expression of vimentin, fibronectin, MMP-2 and MMP-9 [38]. The present study showed reduced nuclear expression of Snail by cirsiliol which was further supported by the retarded expression and activity of MMP-9 in FN-induced melanoma cells.

Twist is a basic helix-loop-helix transcription factor with a strong ability to induce the EMT. Twist can enhance the biological activity of Akt, mainly through interaction with the E-box element in the Akt2 promoter. Akt2 is a crucial factor in the PI3K/Akt signaling pathway, as it regulates cell proliferation, differentiation and survival in physiological and pathological processes [39]. Akt and Twist are involved in a positive feedback loop, which subsequently promoted EMT [10]. Quercetin prevented epidermal growth factor (EGF)-induced EMT via the epidermal growth factor receptor /P13K/Akt/extracellular signal-regulated kinase 1/2 pathway and by suppressing transcription factors, Snail, Slug and Twist, in PC-3 prostate cancer cells [40]. In conformity with the above studies, it was observed that along with suppression of the PI3K/Akt pathway cirsiliol was capable of inhibiting Twist expression in FN-induced B16F10 cells.

NF-κB regulation may occur by a number of ways of which the inhibitor of κB (IκB) negative-feedback loop forms a major pathway. In the present study, though the exact mechanism of inhibition of NF-κB is not known, cirsiliol could possibly interfere with the NF-κB pathway upstream of IκBα phosphorylation or activate the IκBα negative feedback loop which, in turn, caused nuclear export and degradation of NF-κB. In congruence with this, other investigators have reported that activation of NF-κB causes the degradation of the IκBs, which facilitates nuclear translocation of NF-κB complexes. Subsequently, IκBα is newly synthesized which enters the nucleus, dissociates NF-κB from DNA, and escorts it back to the cytoplasm [41]. Moreover, plant polyphenols have been reported as specific blockers of NF-κB transcriptional activity by targeting events in the nucleus. Epigallocatechin-3-gallate (EGCG), a tea polyphenol, was identified as a novel histone acetylase inhibitor. EGCG repressed the recruitment of p300 with an increased binding of the histone deacetylase 3, blocking the acetylation of p65 and NF-κB target gene expression in response to myriad stimuli [42]. Curcumin, an active component of turmeric, was reported to inhibit the phosphorylation and degradation of IκBα and the subsequent translocation of the p65 subunit of NF-κB to the nucleus [43]. NF-κB also participates in the induction of EMT by PI3K/Akt. Ras activates the PI3K/Akt signaling pathway, which consequently reactivates NF-κB to induce the expression of Snail, and this, in turn, leads to the downregulation of E-cadherin expression [27]. Resveratrol, a phytochemical present in grapes, blueberries, raspberries, mulberries, and peanuts, inhibited TGF-β-induced EMT in human pancreatic cancer cell lines, BxPC-3 and Panc-1, via suppression of PI3K/Akt/NF-κB signaling pathways [44]. The present study also elicited that cirsiliol was effective in reducing the nuclear expression of NF-κB p65 subunit in FN-stimulated B16F10 cells. This might have been due to reduced phosphorylation of the PI3K/Akt pathway by cirsiliol.

**Figure 8:**
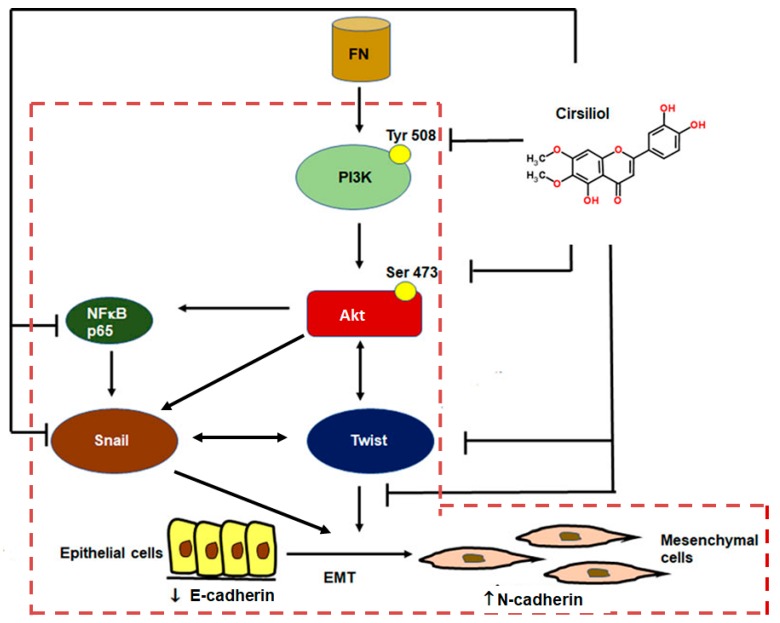
Mechanism of suppression of FN-induced EMT by cirsiliol in B16F10 metastatic melanoma cells. FN activated PI3K/Akt phosphorylation which, in turn, might have upregulated Twist and Snail (independently or via NFκB p65). Snail itself or through interaction with Twist might have suppressed E-cadherin. Cirsiliol was effective in inhibiting phosphorylation of the PI3K/Akt pathway, which in turn caused downregulation N-cadherin and upregulation of E-cadherin. The signaling pathways indicated within the red dotted line box are already established mechanisms. FN, fibronectin; ⊥, pathways inhibited by cirsiliol; → unidirectional mode of action; ↔, bidirectional mode of action.

Prior studies show that FN activates the PI3K/Akt pathway [36], and phosphorylated AktSer473 upregulates NF-κB [45], Snail [27] and Twist [46]. PI3K/Akt directly activates Snail as well as through NF-κBp65-mediated pathway [27,47] and bidirectional mode of interaction exists between Akt and Twist [10] as well as between Snail and Twist [48]. The transcription factors Snail and Twist are responsible for downregulation of E-cadherin and progression of EMT [49]. In this study, we observed that cirsiliol was effective in downregulating the expression of PI3KTyr508, AktSer473, Snail and Twist which altogether might have been responsible for upregulation of E-cadherin, downregulation of N-cadherin and inhibition of FN-induced EMT in B16F10 cells (Figure 8). 

## 4. Materials and Methods

### 4.1. Chemicals

Dulbecco’s modified Eagle’s medium (DMEM) and fetal bovine serum (FBS) were purchased from Gibco Life Technologies Corporation (Grand Island, NY, USA). FN (440 KDa), protease inhibitor cocktail tablets (complete, mini, and - ethylenediaminetetraacetic acid-free), were purchased from Roche (Mannheim, Germany). Gelatin sepharose 4B beads were purchased from GE Healthcare Bio-Sciences AB (Uppsala, Sweden). 3-(4,5-Dimethylthiazol-2-yl)-2-5-diphenyl-2H-tetrazolium bromide (MTT) was purchased from Amresco (Solon, OH, USA). Primary antibodies (monoclonal and polyclonal) to E-cadherin, vimentin, Twist, PI3K, PI3KTyr508, Akt, AktSer473, β-actin and PI3K inhibitor were obtained from Santa Cruz Biotechnology (Santa Cruz, CA, USA). Primary antibodies to N-cadherin, Snail and Slug were purchased from Cell Signaling Technology (Beverly, MA, USA). Secondary antibodies were purchased from Santa Cruz Biotechnologies (Santa Cruz, CA, USA). Super Signal West Femto Maximum sensitivity substrate for enhanced chemiluminescence was purchased from Thermo Scientific (Rockford, IL, USA). RETROscript kit for reverse transcription polymerase chain reaction (RT-PCR) was purchased from Ambion, Life Technologies (Grand Island, NY, USA). TRIzol (for RNA isolation) was purchased from Thermo Fisher scientific (Waltham, MA, USA) and GoTaq DNA polymerase for RT-PCR was procured from Promega (Madison, WI, USA). Primers for E-cadherin, N-cadherin, vimentin matrix MMP-9, MMP-2 and β actin were synthesized by Integrated DNA Technologies, Inc. (Corallville, IA, USA). Costar trans-well plates were purchased from Corning Inc. (Corning, NY, USA).

### 4.2. Extraction and Purification of Cirsiliol

Dried and ground aerial parts of *C. jacea L.* (Asteraceae) were extracted with methanol, and after concentration, the extract was subjected to solvent-solvent partition with *n*-hexane and chloroform. Chromatographic separation, including CC on silica gel and on polyamide, afforded the isolation of cirsiliol (6,7-dimethoxy-5,3’,4’-trihydroxyflavone) in pure form as described previously [17].

### 4.3. Cell Culture

Highly metastatic B16F10 murine melanoma cell line was obtained from National Centre for Cell Sciences (Pune, Maharashtra, India). Cells were cultured and maintained in DMEM-supplemented with 10% FBS, penicillin (100 U/mL) and streptomycin (100 μg/mL) at 37 °C in a humidified atmosphere of 5% CO_2_ incubator.

### 4.4. Cytotoxicity Assay

Cytotoxicity was determined by the MTT assay. Exponentially growing cells (1 × 10^4^) were seeded in 96-well plates. After 24 h of growth, the cells were treated with various concentrations of cirsiliol. Doxorubicin was used as the positive control. Incubation was carried out at 37 °C for 24 h, 48 h and 72 h. A separate lane was also used as a vehicle control, i.e., DMSO; however, apart from the cytotoxicity assays the DMSO concentration was not allowed to exceed 1% for other experiments. MTT solution was added to each well (1.2 mg/mL) and incubated for 4 h. The reaction resulted in the reduction of MTT by the mitochondrial dehydrogenases of viable cells to a purple formazan product. The MTT-formazan product dissolved in DMSO was estimated by measuring absorbance at 570 nm in a multi-wall microplate reader (Infinite M200, TECAN, Mannedrof, Switzerland).

### 4.5. Treatment of Cells 

Cells were allowed to reach 70% confluency before treatment. Four sets of treatment were followed for each experiment: (i) For control, cells (5 × 10^4^) were seeded without any treatment; (ii) Cells (5 × 10^4^) were seeded in pre-coated (cellular FN 20 μg/mL for 2 h at 37 °C) petri dishes and further incubated for 48 h in presence of 5% FBS for induction of EMT; (iii) Cells (5 × 10^4^) were seeded in pre-coated (cellular FN 20 μg/mL for 2 h at 37 °C) petri dishes and treated with subjected to a non-cytotoxic dose of cirsiliol (10 µM) treatment for 48 h (determined from cell viability assay); and (iv) Cells (5 × 10^4^) were seeded and subjected to cirsiliol (10 µM) treatment for 48 h (results not shown since cirsiliol itself did not have any toxicity at this dose).

### 4.6. Colony Formation Assay

Cells were allowed to reach 70% confluency before treatment. Four sets of treatment were followed for each experiment: (i) For control, cells (5 × 10^4^) were seeded without any treatment; (ii) Cells (5 × 10^4^) were seeded in pre-coated (cellular FN 20 μg/mL for 2 h at 37 °C) petri dishes and further incubated for 48 h in the presence of 5% FBS for induction of EMT; (iii) Cells (5 × 10^4^) were seeded in pre-coated (cellular FN 20 μg/mL for 2 h at 37 °C) petri dishes and treated with a non-cytotoxic concentration of cirsiliol (10 µM) for 48 h (based on cell viability assay); and (iv) Cells (5 × 10^4^) were seeded and subjected to cirsiliol (10 µM) treatment for 48 h. Following each treatment, 50,000 cells were again seeded in plate. The cells were maintained in full media for 7 days, following which they were fixed with ethanol, stained with crystal violet stain and observed under bright field microscope (Olympus CX40, Tokyo, Japan) with 400× magnification.

### 4.7. Cell Cycle Analysis

B16/F10 melanoma cells (2 × 10^4^) were harvested, washed with PBS, and resuspended in sterile PBS. Cells were fixed in ice-cold 100% methanol −20 °C for 1 h. The cells were centrifuged at 5000 rpm for 10 min and the cell pellets were suspended in 1 mL of hypotonic buffer (0.5% Triton X-100 in PBS and 0.5 mg/mL RNase), and incubated at 37 °C for 30 min. Subsequently, propidium iodide solution (50 mg/mL) was added, and the mixture was allowed to stand for 1 h in darkness. The Fluorescence emitted from the propidium iodide-DNA complex was quantitated after excitation of the fluorescent dye by a flow cytometer (FACS Calibur, BD Biosciences, San Jose, CA, USA) using Cell Quest software (BD Biosciences, San Jose, CA, USA) equipped with a 488 nm argon laser and a 525 ± 10 nm band pass emission filter. Fluorescence was captured on a FL2H channel with linear amplification. 

### 4.8. Wound Healing Assay

For cell motility determination, wound healing assay was performed following published methods [50]. The monolayer was scratched with a sterile pipette tip in both treated and control sterile petri-dishes followed by washing with serum free culture media (SFCM) for three times to remove cellular debris. The cells were maintained in fresh SFCM and the wound closure was monitored and photographed at 0, 8, 16, and 24 h using an inverted microscope and camera (Nikon, Tokyo, Japan). To quantify the migrated cells, pictures of the initial wounded monolayers were compared with the corresponding pictures of cells at the end of the incubation. Artificial lines fitting the cutting edges were drawn on pictures of the original wounds and overlaid on the pictures of cultures after incubation. Cells that had migrated across the black lines were counted in six random fields from each triplicate treatment.

### 4.9. Transwell Migration Assay

Cell migration was assessed by using modified Boyden’s chamber system [51]. Briefly both treated and control cells were added to Boyden’s invasion chamber (2 × 10^5^ cells/chamber in triplicate) and grown for 24 h in SFCM with the chamber inserted in DMEM containing 2% FBS as chemoattractant. After 24 h, the media was aspirated from the inserts and the membrane was washed thrice with phosphate-buffered saline (PBS). The cells were then fixed with methanol and stained with Gill’s haematoxylin for 10 min. The cells which migrated towards the opposite side of the membrane were viewed under inverted microscope. Cells from at least ten different fields were counted under microscope (Olympus, Tokyo, Japan) equipped with Magnus Pro 3.7 software (Magnusoptics.com) at 40× magnification and the mean was calculated.

### 4.10. Gelatin Zymography

The activity of the gelatinases as detected by zymography are well established protocols for observing the alterations of MMP-2/MMP-9, if any [52,53]. Gelatin zymography was performed to evaluate the activities of matrix metalloproteinases (MMP-2 and MMP-9) in FN-induced cells both in the presence and absence of cirsiliol according to a protocol described previously [54]. 

### 4.11. Extraction and Estimation of Cellular Proteins 

Cell extraction of both treated and control B16F10 cells was performed with radioimmunoprecipitation assay buffer buffer (Tris-HCl 50 mM, pH 7.5; NaCl 150 mM; NP40 10%; sodium deoxycholate 0.5%; sodium dodecyl sulfate (SDS) 0.1%; protease inhibitor cocktail; NaF 0.01%; Na_3_VO_4_ 0.01%) at −80 °C for 1 h. Subsequently, it was followed with centrifugation at 12,000 rpm for 15 min. Cell supernatant was used for protein estimation. Protein estimation was done from the cell lysate using the Lowry’s method [55].

### 4.12. Western Blot Analysis 

Equal amount of total protein (70 μg) of treated and control cells were suspended in Laemmli’s buffer containing β-mercaptoethanol for 5 min at 90 °C. The samples were run on sodium-dodecyl sulphate polyacrylamide gel electrophoresis (SDS-PAGE) (10%) and blotted onto polyvinylidene difluoride membranes. The membranes were blocked using 5% bovine serum albumin in Tris-buffered saline with Tween-20 (TBS-T); 50 mM Tris, 150 mM NaCl, and 0.05% Tween-20), incubated with anti-E-cadherin, anti-N-cadherin, anti-vimentin, anti-Snail, anti-Slug, anti-Twist, anti-Akt, anti-phosphoAktSer473 (p-Akt Ser473), anti-PI3K, anti-phosphoPI3KTyr 508 (p-PI3K Tyr 508) monoclonal and polyclonal antibodies [1:1000 (1 μg antibody in 1 mL buffer) dilution] overnight at 4 °C, washed thrice in TBS-T, and incubated with horse peroxidase coupled secondary antibodies for 90 min at 37 °C and washed thrice with TBST. Bands were visualized using the enhanced chemiluminescence method.

### 4.13. Inhibitor Assay

B16F10 cells (3 × 10^5^ cells/mL) were treated with PI3K inhibitor (LY 294002) (20 mM) for 1 h. The cells were then seeded in plates previously coated with FN (20 µg/mL) as well as without FN. Following this procedure of cell seeding, the cells were treated with cirsiliol (10 µm/48 h) and vehicle control (0.01% DMSO/48 h). The cells were further processed for western blotting.

### 4.14. Immunocytochemistry

B16F10 cells were seeded on coverslips to 70% confluency and then treated with FN alone or FN along with cirsiliol or left untreated for 24 h. After the treatment, the cells were washed with cold PBS and fixed with ice cold methanol for 10 min at −20 °C. The non-specific binding sites were blocked by blocking buffer for 1vh at 37 °C. The cells were then immunolabelled with anti-E-cadherin, anti-N-cadherin, anti-Snail, anti-Twist and anti-NFκB p65 antibody overnight, washed with cold PBS and incubated for 1 h with respective secondary antibodies for 1 h at room temperature. The cells were again washed with PBS followed by 3,3’-diaminobenzidine treatment for 1 h at room temperature and the nucleus were counterstained with hematoxylin for 10 min. The gradation of alcohol was carried out and the coverslips were mounted on glass slides using distyrene, plasticizer and xylene (DPX) and viewed under a microscope (Olympus, Tokyo, Japan) equipped with Magnus Pro 3.7 software at 40× magnification and the mean was calculated.

### 4.15. Semi-Quantitative RT-PCR

Total RNA from FN-induced B16F10 cells treated with or without cirsiliol was isolated using TRIzol reagent and the isolated RNA was quantified spectrophotometrically. cDNA was synthesized from 2 μg of total RNA using RETROscript kit. The cDNAs were amplified with specific primers for E-cadherin (forward-CATGCAGTTCTGCCAGAGGA, reverse-ATCAGAATCAGCAGGGCGAG; NM_009864.3), N-cadherin (forward-TGTGGAGGCTTCTGGTGAAA, reverse-CTTGAAATCTGCTGGCTCGC; NM_007664.5), vimentin (forward-AACGAGTACCGGAGACAGGT; reverse-AGGTCATCGTGATGCTGAGAA; NM_011701.4, MMP-9 (forward-TGAATCAGCTGGCTTTTGTG, reverse-GTGGATAGCTCGGTGGTGTT; NM_013599.4), MMP-2 (forward-AGAGACTGGCTTAGGGC; reverse-GTTGAAGGAAACGAGCGAAG; NM_008610.3), NF-ĸB (forward-CCAAACAGCGAGGCTTCAGA; reverse-ATAGGTCTTCCGGCCCTTCT; NM_001177370.1) and β-actin (forward-GTCCACCTTCCAGCAGATGTG, reverse-GCATTTGCGGTGGACGAT; NM_007393.5). The PCR conditions included an initial denaturation step for 7 min at 95 °C and 35 cycles of 40 s at 95 °C, annealing at 53.9 °C for E-cadherin, 53 °C for N-cadherin and 49.7 °C for MMP-9, 52 °C for β-actin and extension at 72 °C for 30 s. After the last cycle, a final extension was performed at 72 °C for 7 min. β-actin was used as an internal control. 

### 4.16. Quantification of the Results

Bands of zymography, western blots and RT-PCR were quantitated using Image J Launcher (version 1.4.3.67, https://imagej.net/Launcher).

### 4.17. Statistical Analysis

Quantitative results are expressed as mean ± SD. The statistical significance between treated and control groups were determined using the one-way analysis of variance (ANOVA) followed by the Dunnett *t*-test, where *P* value was set at 0.05 to check the statistical difference between groups. The Dunnett *t*-test treats one group as a control and compares all other groups against it. All results were computed and analyzed using the SPSS statistical software package 10.0 (SPSS, Chicago, IL, USA).

## 5. Conclusions

In conclusion, it may be determined that FN-activated PI3K/Akt phosphorylation which, in turn, might have upregulated Twist and Snail via NF-κB p65. Snail might have further stabilized Twist and suppressed E-cadherin. Cirsiliol was effective in suppressing PI3K/Akt/NF-κB pathway which, in turn, caused downregulation of N-cadherin, Snail and Twist and upregulation of E-cadherin (Figure 8). The inhibition of EMT signaling pathway was also reflected in the reduced migratory potential of the FN-stimulated B16F10 cells. Therefore, cirsiliol may be regarded as a promising compound to suppress EMT in the therapeutic management of metastatic melanoma. Nevertheless, further studies, including in vivo experiments, are warranted to understand the full potential of our present findings.

## Supplementary Materials

Supplementary materials can be found at http://www.mdpi.com/1422-0067/20/3/608/s1.
