# Supplementary material for: Cirsiliol Suppressed Epithelial to Mesenchymal Transition in B16F10 Malignant Melanoma Cells through Alteration of the PI3K/Akt/NF-κB Signaling Pathway
Source: Int J Mol Sci. 2019 Jan 31;20(3):608. doi: 10.3390/ijms20030608 (PMC6386903; doi:10.3390/ijms20030608)
Supplement: Supplementary file 1 [file ijms-20-00608-s001.pdf]

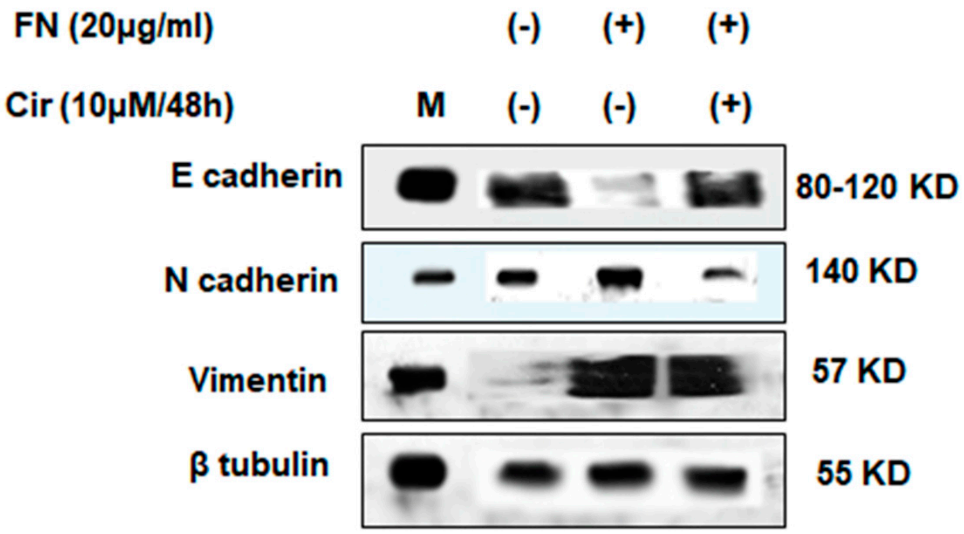

Figure S1. Scanned blots of Figure 4.

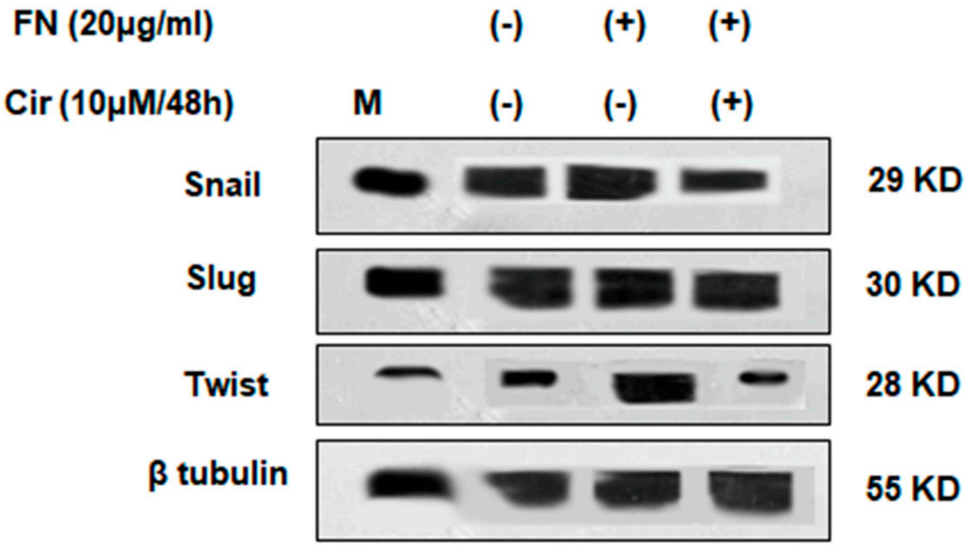

Figure S2. Scanned blots of Figure 5.

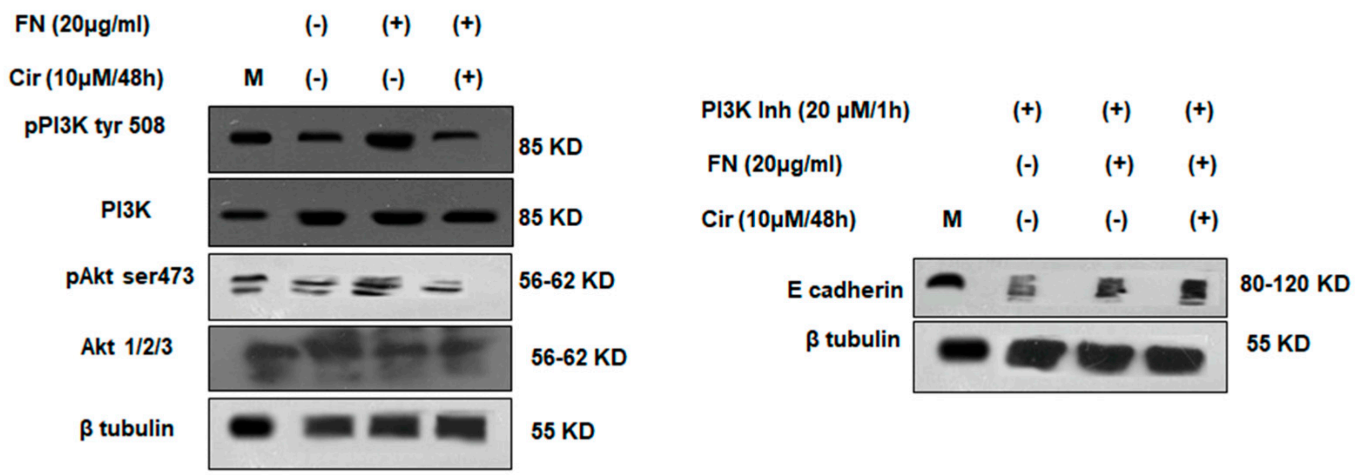

Figure S3. Scanned blots of Figure 6.

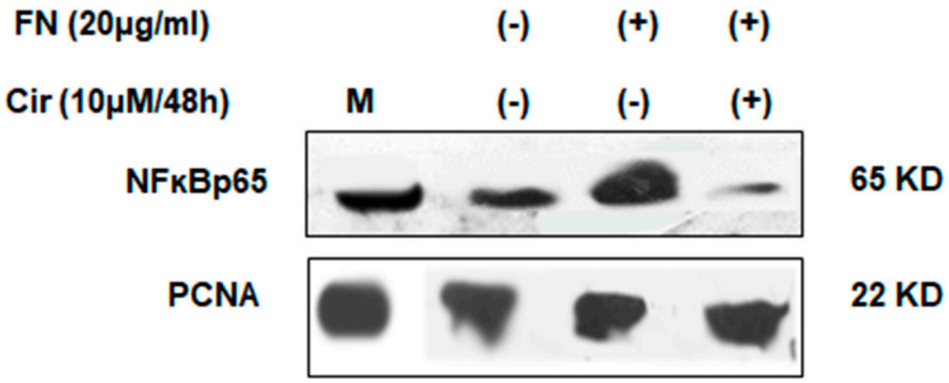

Figure S4. Scanned blots of Figure 7.
